# Design of α/β-Hybrid Peptide Ligands of α4β1 Integrin Equipped with a Linkable Side Chain for Chemoselective Biofunctionalization of Microstructured Materials

**DOI:** 10.3390/biomedicines9111737

**Published:** 2021-11-21

**Authors:** Michele Anselmi, Monica Baiula, Federica Santino, Junwei Zhao, Santi Spampinato, Natalia Calonghi, Luca Gentilucci

**Affiliations:** 1 Department of Chemistry “G. Ciamician”, University of Bologna, Via Selmi 2, 40126 Bologna, Italy; michele.anselmi@iit.it (M.A.); federica.santino2@unibo.it (F.S.); juwzhao@ucdavis.edu (J.Z.); 2 Department of Pharmacy and Biotechnology, University of Bologna, Via Irnerio 48, 40126 Bologna, Italy; monica.baiula@unibo.it (M.B.); santi.spampinato@unibo.it (S.S.); natalia.calonghi@unibo.it (N.C.)

**Keywords:** inflammation, allergy, α4β1 integrin, hybrid α/β peptide, monolayer, zeolite, microcrystals, nanoparticles

## Abstract

Arg-Gly-Asp (RGD)-binding integrins, e.g., αvβ3, αvβ1, αvβ5 integrins, are currently regarded as privileged targets for the delivery of diagnostic and theranostic agents, especially in cancer treatment. In contrast, scarce attention has been paid so far to the diagnostic opportunities promised by integrins that recognize other peptide motifs. In particular, α4β1 integrin is involved in inflammatory, allergic, and autoimmune diseases, therefore, it represents an interesting therapeutic target. Aiming at obtaining simple, highly stable ligands of α4β1 integrin, we designed hybrid α/β peptidomimetics carrying linkable side chains for the expedient functionalization of biomaterials, nano- and microparticles. We identified the prototypic ligands MPUPA-(*R*)-isoAsp(NHPr)-Gly-OH (**12**) and MPUPA-Dap(Ac)-Gly-OH (**13**) (MPUPA, methylphenylureaphenylacetic acid; Dap, 2,3-diamino propionic acid). Modification of **12** and **13** by introduction of flexible linkers at isoAsp or Dap gave **49** and **50**, respectively, which allowed for coating with monolayers (ML) of flat zeolite crystals. The resulting peptide–zeolite MLs were able to capture selectively α4β1 integrin-expressing cells. In perspective, the α4β1 integrin ligands identified in this study can find applications for preparing biofunctionalized surfaces and diagnostic devices to control the progression of α4β1 integrin-correlated diseases.

## 1. Introduction

Integrins are one of the major families of adhesion receptors that mediate cell–cell and cell–extracellular matrix interactions (ECM). These heterodimeric transmembrane proteins are composed by diverse α and β subunits, giving rise in mammals to 24 possible α/β heterodimers [1]. Integrins, expressed on many cell types, regulate fundamental functions, such as adhesion, signaling, and viability, but they are also deeply involved in a variety of diseases, including the initiation and progression of cancer, coronary diseases, inflammatory, and autoimmune pathologies. Consequently, antibodies (Ab) or small molecules that block integrin functions have attracted significant attention as potential drugs [2].

The endogenous ligands of integrins are glycoproteins expressed on the cell surface, e.g., the vascular cell adhesion molecule-1 (VCAM-1) and the intercellular adhesion molecule, or proteins of the ECM, including collagens, fibronectins (FN), vitronectin, laminins, and fibrinogen. The Arg-Gly-Asp (RGD) tripeptide motif was identified in 1984 as a minimal integrin recognition sequence within FN [3]. Later, this tripeptide was found to bind to several integrins, including αvβ3, αvβ1, αvβ5, αvβ6, αvβ8, α5β1, α8β1, expressed on cancer cells, and αIIbβ3, integrin expressed on platelets. Other integrins may interact with their ligands by recognizing different peptide motifs. Hence, peptide or peptidomimetic antagonist ligands that reproduce the integrin-binding sequences can be utilized to interfere with integrin–ligand interactions [4].

**Figure 1:**
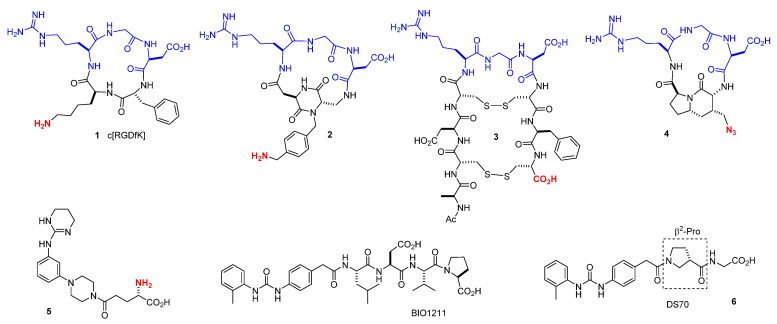
Examples of RGD peptide (**1**–**4**) or peptidomimetic (**5**) integrin ligands recognized by integrins expressed on cancer cells (e.g., αvβ3, α5β1). Functional groups exploitable for peptide conjugation are rendered in red. The α4β1-integrin selective antagonist BIO1211 contains the LDV binding motif and a diphenylurea moiety. The α/β-hybrid DS70 (**6**), designed as an analogue of BIO1211, includes a central β^2^-Pro scaffold.

Unfortunately, most RGD antagonists gave contrasting results and repeatedly failed to demonstrate therapeutic benefits in cancer patients [5]. In search for alternative utilizations [6], the RGD ligands have been conjugated to drugs, drug carrier systems, fluorescent tags, nanoparticles (NPs), materials, etc., for cancer therapy or imaging [7,8], and more recently for innovative applications, such as smart and responsive materials [9,10]. To the purpose, several RGD peptides equipped with suitable linkable side chains have been designed. Examples of cyclic (**1**–**4**) [11,12,13] or peptidomimetic (**5**) [14] RGD ligands are shown in Figure 1. In particular, the cyclopeptide c(RGDfK) (**1**), developed by H. Kessler’s group [15], has become very popular, since the lysine enables the linkage of the peptide to a variety of spacers and materials [16,17,18].

On the other hand, very little attention has been paid so far to the use of integrin-binding motifs different from RGD for material biofunctionalization. In particular, α4β1 integrin recognizes the Leu-Asp-Val-Pro (LDVP) peptide in FN, the Leu-Asp-Thr-Ser (LDTS) sequence in the mucosal addressing cell adhesion molecule-1 (MAdCAM-1), and Ile-Asp-Ser (IDS) in VCAM-1. The α4β1 integrin, also known as CD49d/CD29 or as very late antigen-4 (VLA-4), is expressed on most leukocytes and plays a crucial role in inflammation, allergy, and autoimmune diseases, e.g., multiple sclerosis (MS), but also in cancer development, metastasis, and stem cell mobilization or retention [19]. Targeting α4β1 integrin with the humanized monoclonal antibody (mAb) natalizumab has already proven to be successful for the treatment of highly active relapsing and remitting MS [20]. Several peptidomimetic α4β1 integrin antagonists are currently under investigation for diverse pathologies, such as asthma [21], allergic conjunctivitis [22], age-related macular degeneration (AMD) [23], etc.

In perspective, materials functionalized with α4β1 integrin-binding peptides might consent to design devices for the detection and quantification of leucocytes expressing active α4β1 integrins. The opportunity to detect leukocyte integrin binding for diagnostic applications has been proposed by several authors [24,25,26,27,28,29]. For instance, the binding of the monoclonal antibody N29 to activated β1 integrin on eosinophils was exploited in a patented kit related to asthma control; the integrin amount above a minimum threshold was used as an indicator of decreased lung function [30].

Within this field, very recently we designed a biomaterial constituted by a flat monolayer of microcrystals, capable of reproducing the cell-adhesive multivalency integrin–ligand interaction at the endothelial surfaces in the proximity of the site of inflammation [31]. The monolayers were coated with a peptide ligand derived from the potent α4β1 integrin antagonist BIO1211 (Figure 1), MPUPA-Leu-Asp-Val-Pro-OH, which includes the LDV minimal epitope of the endogenous ligand FN, plus the MPUPA moiety (methylphenylureaphenylacetic acid) at the N-terminus, which strongly increased the affinity for α4 integrins [32]. This microstructured surface allowed significant and selective detection of α4β1 integrin-expressing cells. Unfortunately, the practical utility of this prototypic device is diminished by the poor stability of BIO1211 in physiological conditions, e.g., heparinized blood, plasma and rat liver, lung and intestinal homogenates, where it is metabolized by hydrolytic cleavage of the terminal dipeptide moiety, giving a sequence much less active than the parent compound [33,34].

**Figure 2:**
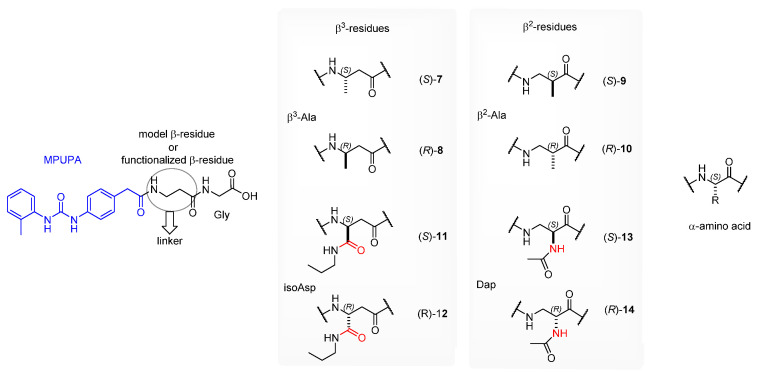
Sketches of candidate α4β1 integrin ligands **7**–**14** based on a hybrid α/β-peptidic structure. In the boxes, model β^3^- or β^2^-aminoacids, i.e., β^3^-Ala and β^2^-Ala, and β^3^- or β^2^-residues carrying side chain functional groups suitable for diverse linker connections, i.e., isoAsp and Dap, respectively, are shown. Functional groups exploitable for peptide conjugation are rendered in red; these are terminated with n-propylamine in **11** and **12**, and with Ac in **13** and **14**.

Aiming at increasing the stability of the peptidic surface, we turned our attention to peptidomimetics composed of α- and β-amino acids [35]. β-Amino acids are classified as β^3^ when the side chain is located adjacent to the β-amino group, or β^2^ when the side chain is adjacent to the carboxylic acid group (Figure 2) [36,37]. In the framework of a program directed to the synthesis of hybrid α/β-peptide integrin ligands [38,39,40,41], we identified the selective α4β1 integrin antagonist DS70 (**6**), containing the (*S*)-pyrrolidine-3-carboxylate (β-Pro) scaffold (Figure 1) [22]. Compound **6** showed a noteworthy affinity for the isolated α4β1 integrin (IC_50_ = 8.3 nM). Consistently, **6** reduced the adhesion of α4β1 integrin-expressing cells to VCAM-1 (IC_50_ = 5.04 nM) or FN (IC_50_ = 4.3 nM). Interestingly, the hybrid structure conferred to this compound a good enzymatic stability, as estimated by incubation in mouse serum [22].

These results prompted us to design analogues of the hybrid α/β-peptide **6** containing diverse β-amino acid cores, equipped with versatile functional groups for expedient connection to linkers (Figure 2) suitable for chemoselective functionalization of biocompatible materials.

## 2. Materials and Methods

### 2.1. General Methods

Standard chemicals were obtained from commercial sources. The purity of intermediates and final products were analyzed by reverse-phase (RP) HPLC, performed on Agilent 1100 series apparatus (Agilent, Technologies, Waldbronn, Germany), equipped with a RP column Phenomenex (Torrance, CA, USA) No 00D-4439-Y0 Gemini 3 μm C18 110 Å, LC column 100 × 3.0 mm; diode-array detector (DAD) λ = 210 nm and 254 nm; mobile phase: from 9:1 H_2_O/acetonitrile (ACN) containing 0.1% formic acid to 2:8 H_2_O/ACN containing 0.08% formic acid in 20 min, flow rate 1.0 mL min^−1^. Semipreparative RP HPLC utilized an Agilent 1100 apparatus, equipped with a RP column ZORBAX No 977150-102 Eclipse XDB C18 PrepHT cartridge 21.2 × 150 mm, 7 μm (Agilent Technologies, Santa Clara, CA, US); mobile phase: from 8:2 H_2_O/ACN/0.1% trifluoroacetic acid (TFA) to 100% CAN/0.1% TFA in 10 min, flow rate 12 mL min^−1^. Electrospray ionization MS analysis was performed on a HP mass spectrometer MSD 1100 detector (Agilent Technologies) with single quadrupole. ^1^H NMR analysis was performed on a Varian Gemini 400 MHz apparatus (Agilent Technologies); peptide samples were dissolved in DMSO-d6 to the final concentration of 0.01 M and analyzed in 5 mm tubes at rt. Water suppression required the PRESAT presaturation procedure. Chemical shifts (δ) are expressed as p.p.m., using DMSO as an internal standard (δH = 2.50 p.p.m.). The assignment of all resonances was based on two-dimensional gradient selected correlation spectroscopy experiments (gCOSY).

### 2.2. Peptide Synthesis

Boc-β^3^-homoAla-Gly-OBn (*S*)-**33**, (*R*)-**34**. A mixture of (*S*)-**21** or (*R*)-**22** (0.25 mmol, Supplementary Materials), hydroxybenzotriazole (HOBt, 0.4 mmol), 1-ethyl-3-(3-dimethylaminopropyl) carbodiimide**^.^**HCl (EDC, 0.4 mmol), and TEA (0.8 mmol), in 4:1 DCM/DMF (5 mL), was stirred at rt under N_2_ atmosphere. After 15 min, H-Gly-OBn**^.^**TFA (0.3 mmol) was added, and the mixture was stirred under N_2_ at rt overnight. Then, the solvent was evaporated at reduced pressure, and the residue was diluted with EtOAc (30 mL). The suspension was washed with 0.1 M HCl (5 mL), and a saturated solution of NaHCO_3_ (5 mL). The organic layer was dried over Na_2_SO_4_, filtered, and the solvent was evaporated at reduced pressure. Purification by flash chromatography over silica gel (eluent: cyclohexane/EtOAc 60:40) allowed the isolation of (*S*)-**33** (73%) or (*R*)-**34** (63%) as a white solid. *Rf* = 0.23 in cyclohexane/EtOAc 50:50. ESI-MS *m*/*z* calculated for (C_18_H_26_N_2_O_5_Na)^+^: 373.2, found: 373.0 (M+Na)^+^.

H-β^3^-HomoAla-Gly-OBn.TFA (*S*)-**35**, (*R*)-**36**. The peptide (*S*)-**33** or (*R*)-**34** (0.19 mmol) was treated with 25% TFA in DCM (5 mL) at 0 °C, and the mixture was shaken at rt for 1 h. Then, the solvent was evaporated at reduced pressure, and ice-cold Et_2_O was added to precipitate the peptide(*S*)-**35** or (*R*)-**36** as TFA salt in quantitative yield. The salt was collected by filtration, and used for the next step without further purifications. ESI-MS *m*/*z* calcd. for [C_13_H_19_N_2_O_3_]^+^: 251.1, found: 251.2 [M + H]^+^.

MPUPA-β^3^-homoAla-Gly-OBn (*S*)-**37**, (*R*)-**38**. The intermediate (*S*)-**35** or (*R*)-**36** (69 mg, 0.19 mmol) was coupled in turn with MPUPA (0.2 mmol) in the presence of the activating agents EDC/HOBt/TEA (0.4/0.4/0.8 mmol), under the same conditions described above. After the usual work up, the resulting crude residue was purified by flash chromatography over silica gel (eluent: cyclohexane/EtOAc 40:60), giving (*S*)-**37** (85%) or (*R*)-**38** (96%) as a white solid. *Rf* = 0.18 in EtOAc/MeOH 95:5). ESI-MS *m*/*z* calculated for (C_29_H_33_N_4_O_5_)^+^: 517.2, found: 517.0 (M + H)^+^.

MPUPA-β^3^-homoAla-Gly-OH (*S*)-**7**, (*R*)-**8**. The protected (*S*)-**37** or (*R*)-**38** (0.18 mmol) was subjected to catalytic hydrogenation over 10% Pd/C (*w*/*w*) in dry EtOH (20 mL) at rt for 24 h. The mixture was filtered over Celite^®^, the filter was washed with EtOH, and the solvent was evaporated at reduced pressure. The residues were purified by semipreparative RP HPLC (general methods) yielding the final (*S*)-**7** (76%, 93% pure) or (*R*)-**8** (87%, 95% pure) as a white solid. Purity was assessed by analytical RP HPLC (general methods); *Rt* = 4.4 min. ^1^H NMR (600 MHz, DMSO-*d*_6_) δ 8.95 (s, 1H, ureaNH), 8.20 (dd, *J* = 6.0, 5.4 Hz, 1H, GlyNH), 7.90 (d, *J* = 7.8 Hz, 1H, β^3^-AlaNH), 7.88 (s, 1H, ureaNH), 7.83 (d, *J* = 8.4 Hz, 1H, ArH_6_), 7.36 (d, *J* = 8.4 Hz, 2H, ArH_2′6′_), 7.20–7.09 (m, 4H, ArH_3,5_ + ArH_3′5′_), 6.93 (dd, *J* = 7.6, 7.2 Hz, 1H, ArH_4_), 4.05 (dd, *J* = 14.4, 7.8 Hz, 1H, β^3^-AlaHβ), 3.72 (dd, *J* = 17.4, 6.0 Hz, 2H, GlyHα), 3.30 (s, 2H, PhCH_2_), 2.35 (dd, *J* = 13.8, 5.4 Hz, 1H, β^3^-AlaHα), 2.23 (s, 3H, ArCH_3_), 2.18 (dd, *J* = 13.8, 8.4 Hz, 1H, β^3^-AlaHα), 1.05 (d, *J* = 6.6 Hz, 3H, CH_3_). ^13^C NMR (150 MHz, DMSO-*d*_6_) δ 172.0, 171.1, 170.1, 153.4, 138.8, 138.1, 130.8, 130.4, 129.9, 128.1, 126.8, 123.3, 121.6, 118.6, 43.0, 42.5, 41.2, 40.6, 20.6, 18.5. ESI-MS *m*/*z* calculated for (C_22_H_27_N_4_O_5_)^+^: 427.2, found: 427.0 (M + H)^+^.

MPUPA-β^2^-homoAla-OMe (*S*)-**40**, (*R*)-**41**. A stirred solution of MPUPA (1.0 mmol), HOBt (1.3 mmol), 2-(1H-benzotriazole-1-yl)-1,1,3,3-tetramethylaminium tetrafluoroborate (TBTU, 1.3 mmol), and N,N-diisopropylethylamine (DIPEA, 2.6 mmol) in 4:1 DCM/DMF (8 mL) was stirred at rt under N_2_. After 15 min, the amine (*S*)-**27** or (*R*)-**28** (1.0 mmol, Supplementary Materials) was added, and the mixture was stirred at rt under N_2_ overnight. Then, the mixture was concentrated at reduced pressure, and the residue was diluted with EtOAc (30 mL). The mixture was washed with 0.1 M HCl (5 mL) and a saturated solution of NaHCO_3_ (5 mL). The organic layer was dried over Na_2_SO_4_ and the solvent evaporated at reduced pressure, affording the intermediate (*S*)-**40** (71%) or (*R*)-**41** (61%), which was used for the next step without further purifications. ESI-MS *m*/*z* calculated for (C_21_H_26_N_3_O_4_)^+^: 384.2, found: 384.2 (M + H)^+^.

MPUPA-β^2^-homoAla-OH (*S*)-**42**, (*R*)-**43**. The hydrolysis of the methyl ester of (*S*)-**40** or (*R*)-**41** (0.12 mmol) was carried out by treatment with LiOH (0.2 mmol) in 2:1 THF/H_2_O (*v*/*v*, 4 mL) at 0 °C. The reaction was stirred at rt and the progress was checked by TLC. After 3 h, the pH of the mixture was lowered to 4 at 0 °C by adding 0.1 M HCl dropwise, then the mixture was concentrated at reduced pressure. The crude intermediate (*S*)-**42** or (*R*)-**43** was obtained in quantitative yield and was used for the next step without further purification. ESI-MS *m*/*z* calculated for (C_20_H_24_N_3_O_4_)^+^: 370.2, found: 370.2 (M + H)^+^.

MPUPA-β^2^-homoAla-Gly-OtBu (*S*)-**44**, (*R*)-**45**. The intermediate **42** or **43** (0.12 mmol) was coupled with H-Gly-OtBu**^.^**HCl (0.12 mmol) in the presence of HOBt/TBTU/DIPEA (0.15/0.15/0.2 mmol), as reported above. After the usual work up, the residue was subjected to purification by semipreparative RP HPLC, giving (*S*)-**44** (50%, 96% pure) or (*R*)-**45** (54%, 94% pure) (general methods). Purity was assessed by analytical RP HPLC (general methods). ESI-MS *m*/*z* calculated for (C_26_H_35_N_4_O_5_)^+^: 483.3, found: 483.2 (M + H)^+^.

MPUPA-β^2^-homoAla-Gly-OH (*S*)-**9**, (*R*)-**10**. The *t*Bu-protecting group was removed from (*S*)-**44** or (*R*)-**45** (1.0 mmol) by treatment with 50% TFA in DCM (3 mL) at 0 °C, and the mixture was shaken at rt for 3 h. Then, the solvent was removed at reduced pressure, and the peptide (*S*)-**9** (95% pure) or (*R*)-**10** (94% pure) was precipitated as a white solid from ice-cold Et_2_O in nearly quantitative yield. Purity was assessed by analytical RP HPLC (general methods); *Rt* = 4.5 min. ^1^H-NMR (400 MHz, DMSO-*d*_6_) δ 8.93 (s, 1H, ureaNH), 8.17 (dd, *J* = 6.0, 5.6 Hz, 1H, GlyNH), 7.90 (d, *J* = 6.0 Hz, 1H, β^2^-AlaNH), 7.87 (s, 1H, ureaNH), 7.83 (d, *J* = 8.4 Hz, 1H, ArH_6_), 7.36 (d, *J* = 8.4 Hz, 2H, ArH_2′,6′_), 7.20–7.09 (m, 4H, ArH_3,5_ + ArH_3′,5′_), 6.93 (dd, *J* = 7.6, 7.2 Hz, 1H, ArH_4_), 3.73 (dd, *J* = 17.6, 6.0 Hz, 2H, GlyCH_2_), 3.35–3.30 (m, 2H, PhCH_2_), 3.11 (dd, *J* = 7.2, 5.6 Hz, 2H, β^2^-AlaHβ), 2.55–2.50 (m, 1H, β^2^-AlaCHα), 2.23 (s, 3H, ArCH_3_), 0.97 (d, *J* = 6.0 Hz, 3H, CH_3_). ^13^C-NMR (100 MHz, DMSO-*d*_6_) δ 174.6, 171.4, 170.5, 152.7, 138.1, 137.4, 130.2, 129.7, 129.3, 127.4, 126.1, 122.6, 121.0, 118.0, 41.9, 41.7, 40.5, 17.9, 15.4. ESI-MS *m*/*z* calculated for (C_22_H_27_N_4_O_5_)^+^: 427.2, found: 427.2 (M + H)^+^. 

MPUPA-isoAsp(NHPr)-Gly-OH (*S*)-**11**, (*R*)-**12**. Wang resin preloaded with Fmoc-Gly (loading capacity 0.6 mmol/g, 0.15 mmol) was swollen in DMF for 10 min while shaking into a reactor equipped with a frit. Fmoc cleavage was carried out with 20% piperidine (Pip) in DMF (2 × 5 mL), while shaking at rt for 25 min. The resin was filtered and washed alternatively with DMF (3 × 4 mL), MeOH (3 × 5 mL), and DCM (3 × 5 mL).

Fmoc-isoAsp(NHPr)-OH (*S*)-**31** or (*S*)-**32** (0.3 mmol, Supplementary Materials) was pre-activated with HOBt (0.36 mmol) in DMF (4 mL) in a separate vial, and after 15 min the mixture was transferred into the reactor, followed by dicyclohexylcarbodiimide (DCC, 0.36 mmol). The suspension was gently shaken at rt for 3 h, then the resin was washed alternatively with DMF (3 × 4 mL), MeOH (3 × 5 mL), and DCM (3 × 5 mL). The efficacy of the coupling reaction was monitored by the Kaiser test.

The Fmoc peptidyl resin was deprotected by treatment with Pip/DMF (2 × 4 mL), as reported above. MPUPA (0.3 mmol) was introduced in the presence of HOBt/DCC (0.36 mmol each) as the activating agents, under the same conditions described above.

After the usual work up, the cleavage of the peptide from the resin was carried out using a mixture of TFA/triisopropylsilane (TIPS)/H_2_O/PhOH (78:10:10:2 *v*/*v*/*v*/*v*, 10 mL) for 2.5 h at rt. The mixture was filtered, and the resin was washed with 1:1 Et_2_O/DCM (*v*/*v*, 10 mL) containing 1% of TFA. The collected filtrates were concentrated at reduced pressure at rt, and the crude peptide was triturated in Et_2_O. Purification by semipreparative RP HPLC (general methods) gave peptide (*S*)-**11** (82%, 98% pure) or (*R*)-**12** (65%, 96% pure). Purity was assessed by analytical RP HPLC (general methods); *Rt* = 5.7 min. ^1^H NMR (400 MHz, DMSO-*d*_6_) δ 8.94 (s, 1H, ureaNH), 8.16 (t, *J* = 6.0 Hz, 1H, GlyNH), 8.11 (d, *J* = 8.0 Hz, 1H, isoAspNH), 7.87 (s, 1H, ureaNH), 7.83 (d, *J* = 8.4 Hz, 1H, ArH_6_), 7.67 (dd, *J* = 6.0, 5.2 Hz, 1H, CONH), 7.36 (d, *J* = 8.4 Hz, 2H, ArH_2′6′_), 7.20–7.09 (m, 4H, ArH_3,5_ + ArH_3′5′_), 6.93 (dd, *J* = 7.6, 7.2 Hz, 1H, ArH_4_), 4.52 (dd, *J* = 14.4, 7.2 Hz, 1H, isoAspHα), 3.74 (dd, *J* = 17.6, 5.6 Hz, 1H, GlyHα), 3.70 (dd, *J* = 17.6, 5.6 Hz, 1H, GlyHα), 3.39 (s, 2H, PhCH_2_), 2.98 (m, 2H, propyl-CH_2_), 2.56 (dd, *J* = 14.8, 6.4 Hz, 1H, isoAspHβ), 2.45 (dd, *J* = 15.2, 7.6 Hz, 1H, isoAspHβ), 2.23 (s, 3H, ArCH_3_), 1.42–1.31 (m, 2H, propyl-CH_2_), 0.79 (t, *J* = 7.4 Hz, 3H, propyl-CH_3_). ^13^C NMR (100 MHz, DMSO-*d*_6_) δ 171.2, 170.5, 170.2, 169.6, 152.6, 138.2, 137.4, 130.1, 129.5, 129.4, 127.4, 126.1, 122.6, 120.9, 117.9, 49.8, 41.4, 40.6, 40.4, 37.5, 22.2, 17.9, 11.2. ESI-MS *m*/*z* calculated for (C_25_H_32_N_5_O_6_)^+^: 498.2, found: 498.0 (M + H)^+^. 

MPUPA-Dap(Ac)-Gly-OH (*S*)-**13**, (*R*)-**14**. Wang resin preloaded with Fmoc-Gly (0.6 mmol/g loading capacity, 0.24 mmol) was utilized as described above. After Fmoc cleavage with 20% Pip/DMF (2 × 4 mL), as reported above, coupling reaction with (*S*)- or (*R*)-Fmoc-Asn-OH (0.6 mmol) was carried out in the presence of HOBt/DCC (0.6 mmol) in DMF (5 mL), as described above.

After the usual work up, the resin was washed twice with a mixture of THF/DMF/H_2_O (2:2:1 *v*/*v*/*v*) and subsequently swollen with the same mixture. After 30 min, a solution of PIFA (0.3 mmol) in THF/DMF/H_2_O (2:2:1) was added to the resin followed by pyridine (0.3 mmol) and the mixture was gently stirred at rt for 3 h. Then, the resin was washed with DMF (3 × 5 mL), DMF/DIPEA (9:1), and DMF (3 × 5 mL). The at least partially effective rearrangement was confirmed by a positive Kaiser test.

The peptide sequence was elongated with MPUPA (0.6 mmol) using the same coupling agents as before, HOBt/DCC (0.6 mmol) in DMF (5 mL). The central Dap(Fmoc) residue was deprotected with 20% Pip/DMF (2 × 4 mL), giving the intermediates (*S*)-**46** or (*R*)-**47**. Acetylation of the free amine at Dap of (*S*)-**46** or (*R*)-**47** was performed with Ac_2_O (5.0 eq) and pyridine (5.0 eq) in DCM, and the resulting suspension was shaken at rt for 1 h. 

Cleavage from the resin was carried out using TFA/TIPS/H_2_O (80:10:10 *v*/*v*/*v*, 10 mL) at rt for 2.5 h. The reaction was filtered, the resin was washed with Et_2_O, and the mixture was concentrated at reduced pressure. The residue was transferred into a centrifugation vial and the peptide was precipitated with ice-cold Et_2_O. The crude peptide was purified by semipreparative RP HPLC (general methods) to give (*S*)-**13** (70%, 96% pure) or (*R*)-**14** (58%, 95% pure) as a white powder. Purity was assessed by analytical RP HPLC (general methods); *Rt* = 3.4 min. ^1^H-NMR (400 MHz, DMSO-*d*_6_) δ 8.97 (s, 1H, ureaNH), 8.19 (dd, *J* = 6.4, 5.2 Hz, 1H, GlyNH), 7.97–7.91 (m, 2H, DapNHα + DapNHβ), 7.89 (s, 1H, ureaNH), 7.81 (d, *J* = 8.0 Hz, 1H, ArH_6_), 7.36 (d, *J* = 8.0 Hz, 2H, ArH_2′6′_), 7.19–7.10 (m, 4H, ArH_3,5_ + ArH_3′5′_), 6.93 (dd, *J* = 7.6, 7.2 Hz, 1H, ArH_4_), 4.37 (dd, *J* = 13.6, 8.0 Hz, 1H, DapHα), 3.75-3.68 (m, 2H, GlyHα), 3.41–3.33 (m, 3H, PhCH_2_ + DapHβ), 3.31–3.20 (m, 1H, DapHβ), 2.23 (s, 3H, ArCH_3_), 1.83 (s, 3H, Ac). ^13^C NMR (100 MHz, DMSO-*d*_6_) δ 170.9, 170.3, 169.4, 152.7, 138.2, 137.5, 130.1, 129.5, 129.4, 127.4, 126.1, 122.6, 120.9, 117.9, 52.5, 41.6, 40.6, 22.6, 17.9. ESI-MS *m*/*z* calculated for (C_23_H_28_N_5_O_6_)^+^: 470.2, found: 470.2 (M + H)^+^.

MPUPA-(R)-isoAsp(Hda)-Gly-OH.TFA **49**. Peptide **49** was prepared in SPPS, as reported for peptide **12**, on a Wang resin preloaded with Fmoc-Gly (0.15 mmol). In brief, Fmoc cleavage was carried out with 20% Pip/DMF. Then, Fmoc-(*R*)-isoAsp(Boc-Hda)-OH (**48**, 0.3 mmol, Supplementary Materials) and MPUPA (0.3 mmol), were coupled in sequence in the presence of HOBt/DCC (0.36 mmol each). After the usual work up, the cleavage of the peptide from the resin was performed with TFA/TIPS/H_2_O/PhOH (78:10:10:2 *v*/*v*/*v*/*v*, 10 mL). Purification by semipreparative RP HPLC (general methods) gave **49** (63%, 97% pure). Purity was assessed by analytical RP HPLC (general methods); *Rt* = 1.9 min. ^1^H NMR (400 MHz, DMSO-*d*_6_) δ 9.09 (s, 1H, ureaNH), 8.68 (br.t, 1H, GlyNH), 8.14 (d, *J* = 8.0 Hz, 1H, isoAspNH), 8.02–8.12 (m, 3H, NH_3_^+^), 8.02 (s, 1H, ureaNH), 7.82 (d, *J* = 8.4 Hz, 1H, ArH_6_), 7.62 (br.t, 1H, CONH), 7.37 (d, *J* = 8.4 Hz, 2H, ArH_2′6′_), 7.10–7.18 (m, 4H, ArH_3,5_ + ArH_3′5′_), 6.93 (t, *J* = 7.2 Hz, 1H, ArH_4_), 4.50 (m, 1H, isoAspHα), 3.73 (d, *J* = 5.6 Hz, 2H, GlyHα), 3.58–3.62 (m, 2H, CH_2_), 3.30–3.40 (m, 4H, PhCH_2_ + CH_2_), 2.70–2.78 (m, 2H, CH_2_), 2.60 (dd, *J* = 14.8, 6.4 Hz, 1H, isoAspHβ), 2.45 (dd, *J* = 15.2, 7.6 Hz, 1H, isoAspHβ), 2.23 (s, 3H, ArCH_3_), 1.63 (m, 1H, CH_2_), 1.55–1.55 (m, 3H, CH_2_), 1.35–1.40 (m, 2H, CH_2_), 1.20–1.25 (m, 2H, CH_2_). ^13^C NMR (100 MHz, DMSO-d6) δ 171.6, 171.2, 170.9, 170.1, 153.2, 137.9, 130.6, 129.8, 126.5, 123.0, 121.6, 118.3, 50.3, 44.1, 41.1, 38.7, 29.1, 26.1, 25.9, 22.6, 22.0, 18.2, 15.6. ESI-MS *m*/*z* calculated for (C_28_H_39_N_6_O_6_)^+^: 555.3, found: 555.4 (M + H)^+^.

MPUPA-Dap(Ahx)-Gly-OH.TFA **50**. The MPUPA-(*S*)-Dap-Gly peptidyl resin **46** (0.2 mmol, estimated on the basis of resin load), prepared as described, was functionalized at the free α-amino group of Dap with Boc-Ahx-OH, preactivated with DCC/HOBt (0.5 mmol) in DMF (5 mL). Eventually, the Boc-protected peptidyl resin was treated with TFA/TIPS/H_2_O (80:10:10 *v*/*v*/*v*, 10 mL) at rt for 2.5 h. After the same work up procedure reported previously, the crude peptide was purified by semipreparative RP HPLC (general methods) to give (*S*)-**15** (66%, 97% pure). Purity was assessed by analytical RP HPLC (general methods); *Rt* =1.9 min. ^1^H NMR (400 MHz, DMSO-*d*_6_) δ 9.57 (s, 1H, ureaNH), 8.31 (s, 1H, ureaNH), 8.23 (t, *J* = 5.9 Hz, 1H, GlyNH), 7.99 (t, *J* = 5.9 Hz, 1H, DapNHβ), 7.96–7.83 (m, 4H, DapNHα + NH_3_^+^), 7.81 (d, *J* = 8.0 Hz, 1H, ArH_3_), 7.39 (d, *J* = 8.2 Hz, 2H, ArH_3,5_), 7.08–7.18 (m, 4H, ArH_2,6_ + ArH_4,5_), 6.92 (t, *J* = 7.4 Hz, 1H, ArH_6_), 4.38 (m, 1H, DapHα), 3.74 (d, *J* = 5.8 Hz, 2H, GlyHα), 3.38 (ddd, *J* = 13.5, 5.9, 4.8 Hz, 1H, DapHβ), 3.34 (s, 2H, PhCH_2_), 3.28 (ddd, *J* = 13.5, 7.1, 5.9 Hz, 1H, DapHβ), 2.72–2.77 (m, 2H, AhxHε), 2.25 (s, 3H, PhCH_3_), 2.10 (t, *J* = 7.5 Hz, 2H, AhxHα), 1.52–1.57 (m, 2H, AhxHδ), 1.42–1.48 (m, 2H, AhxHβ), 1.22–1.30 (m, 2H, AhxHγ). ^13^C NMR (100 MHz, DMSO-d6) δ 172.3, 171.2, 171., 170.51, 153.0, 138.6, 137.6, 130.2, 129.4, 129.3, 127.7, 126.1, 122.6, 121.2, 118.3, 117.9, 115.3, 52.7, 41.7, 40.8, 40.6, 38.7, 35.0, 26.8, 25.5, 24.5, 18.2. ESI-MS *m*/*z* calculated for (C_27_H_37_N_6_O_6_)^+^: 541.3, found: 541.2 (M + H)^+^.

### 2.3. Biochemical Characterization

#### 2.3.1. Cell Culture

All cell lines were purchased from the ATCC (Rockville, MD, USA). Jurkat E6.1 cells (expressing α4β1) were maintained as a stationary suspension culture in RPMI-1640 (Life Technologies, Carlsbad, CA, USA) supplemented with 2 mM glutamine and 10% FBS (fetal bovine serum). HEK293 cells were routinely cultured in EMEM (Lonza, Basel, Switzerland) enriched with 10% FBS, L-glutamine and nonessential amino acids. Cells were kept at 37 °C under a 5% CO_2_ humidified atmosphere.

#### 2.3.2. Cell Adhesion Assays on the Endogenous Ligand FN

The adhesion assays were performed as previously described [22]. Briefly, black 96-well plates (Corning Costar, Celbio, Milan, Italy) were coated with FN (10 μg/mL). Cells were counted, stained with CellTracker green CMFDA (12.5 μM, 30 min at 37 °C, Life Technologies), and after three washes with 1% BSA (bovine serum albumin) in HBSS (Life Technologies) were preincubated with increasing concentrations of the new peptide ligands (10^−10^–10^−4^ M) or with the vehicle (methanol) for 30 min at 37 °C. Afterwards, cells were plated (500,000/well) on coated wells and incubated for 30 min at 37 °C. After three washes, adhered cells were lysed with 0.5% Triton X-100 in PBS (30 min at 4 °C) and fluorescence was measured (Ex485 nm/Em535 nm) in an EnSpire Multimode Plate Reader (PerkinElmer, Waltham, MA, USA).

The number of adherent cells was obtained by comparison with a standard curve prepared in the same plate using diverse concentrations of cells. Experiments were carried out in quadruplicate and repeated at least three times. Data analysis and IC_50_ values were calculated using GraphPad Prism 6.0 (GraphPad Software, San Diego, CA, USA).

#### 2.3.3. Enzymatic Stability

Peptides **12**, **13**, and BIO1211, were dissolved in Tris buffer (pH 7.4) to give a concentration of 10 mM, and 10 µL aliquots of these stock solutions were added to 190 µL of mouse serum. Incubations were carried out at 37 °C. Aliquots of 20 µL were sampled over 180 min from the mixtures and the samples were diluted with 90 µL of glacial ACN for precipitating proteins. The mixtures were diluted with 90 µL of 0.5% AcOH and centrifuged (13,000× *g*, 15 min). The supernatants were collected and the amount of intact peptides was determined by RP HPLC. The experiments were performed in triplicate and repeated three times.

### 2.4. Preparation of the Biofunctionalized Surfaces 49–Zeolite MLs/50–Zeolite MLs, and of the Negative References BA–Zeolite MLs/49-Plates

A mixture of TEA (10 µL) and peptide **49** or **50** (1.5 mg) or n-butylamine (BA, 0.3 mg) in DMF (1 mL) was dissolved by sonication for 5 min. The IC–zeolite MLs (Supplementary Materials) were immersed in the solution and heated at 40 °C. After 3 h, the plates were removed and washed with DMF (5 mL) and EtOH (5 mL), then the MLs were air dried.

To prepare the negative control **49**-plates, bare silica plates were reacted with ICPTES, and the resulting IC-plates were treated with peptide **49**, under the same conditions utilized for the preparation of **49**–zeolite MLs.

### 2.5. Cell Adhesion on 49–Zeolite MLs/50–Zeolite MLs/BA–Zeolite MLs/49-Plates

Jurkat or HEK293 (10^6^ cells/mL) cells were suspended in 1% BSA in PBS in the presence of the fluorescent dye CMFDA (6.25 µM) and incubated for 20 min at 37 °C while gently shaken. At the end of the incubation, the cells were washed three times with 1 mL of 1% BSA in PBS.

The peptide MLs were washed with PBS solution, then they were placed in a 6-well plate (growth area 8.87 cm^2^) and seeded with 5 × 10^5^ Jurkat or HEK-293 cells. Each well was filled with PBS (final volume, 1 mL), and the plates were incubated for 15 min. Afterwards, each well was washed twice with PBS and the sample fixed with 3% PFA for 10 min at RT. The plates were washed twice with 0.1% glycine in PBS, and each monolayer was embedded in Mowiol and analyzed with a confocal laser-scanning microscope. 

### 2.6. Confocal Microscopy

For visualization of the DXP-loaded zeolites in red, fluorescence was excited with a λ = 543 nm laser and detected at 650 nm; CMFDA-stained cells were visualized in green by excitation at 488 nm and detected at λ = 530 nm. Images were quantified by ImageJ software version 1.52 t, NIH, USA. The number of cells adherent to a surface of 0.0025 cm^2^ was determined three times, and the average was expressed as cell.cm^−2^ ± S.D. Statistical comparison was performed using a Student’s *t*-test; *p* < 0.05 was considered significant.

## 3. Results

### 3.1. Peptide Design and Synthesis

Moving from the α/β-hybrid lead **6** (Figure 1), we designed a series of analogues (Figure 2) containing diverse β-amino acid cores, while maintaining glycine as the C-terminal residue and the diphenylurea moiety MPUPA (Figure 2). The mini-library of β-residues included model homo β^2^- or β^3^-amino acids in (*S*)- or (*R*)-configuration, i.e., β^3^-Ala (peptides **7**, **8**) or β^2^-Ala (**9**, **10**), as well as (*S*)- or (*R*)-configured β^3^- or β^2^-residues carrying side chain functional groups exploitable for expedient connections, i.e., isoaspartate (isoAsp, **11**, **12**), or 2,3-diamino propionic acid (Dap, **13**, **14**), respectively.

**Scheme 1:**
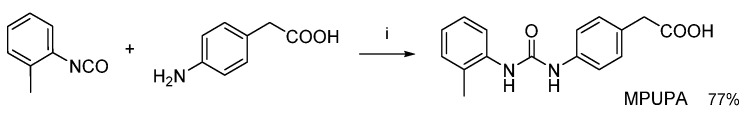
Synthetic procedure for MPUPA. Reagents and conditions: (i) DMF, rt, 3 h.

The MPUPA moiety was prepared (Supplementary Materials) by reaction between o-tolyl isocyanate and 2-(4-aminophenyl)acetic acid (Scheme 1).

#### 3.1.1. Synthesis of β-Amino Acids

**Scheme 2:**
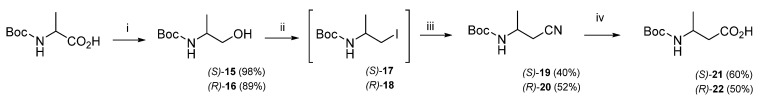
Synthetic procedure for (*S*)- or (*R*)-Boc-β^3^-homoAla. Reagents and conditions: (i) NMM, ethyl chloroformate, THF, 0 °C, rt, 15 min; then, NaBH_4(aq)_ 0 °C, rt, 10 min; (ii) PPh_3_, I_2_, imidazole, DCM, reflux, 3 h; (iii) KCN, DMSO, 60 °C, 4 h; (iv) 1 M KOH/EtOH/ (1:1 *v*/*v*), 90 °C, 3 h, then 1 M KHSO_4_.

Many β-amino acids are commercially available in diverse protected forms. Nevertheless, we opted for in-house preparations, potentially exploitable for preparing a variety of (*S*)- or (*R*)-, natural or unnatural β-residues. β^3^-amino acids can be obtained by direct Arndt–Eistert homologation of the corresponding α-amino acids. In several cases, this procedure was found to be poorly efficient and not suitable for large scale preparation [42]. Therefore, we utilized the scalable procedure reported by Caputo et. al. (Scheme 2) [43].

The β-amino alcohol (*S*)-**15** or (*R*)-**16** was obtained from (*S*)- or (*R*)-Boc-Ala-OH, respectively, by reduction of the mixed anhydride with NaBH_4_. The β-amino alcohol was then converted into the corresponding β-amino iodide (*S*)-**17** or (*R*)-**18** by means of triphenylphosphine/iodide complex. 

Due to poor stability under the common separation and purification procedures, the iodoamino intermediate was used directly without isolation. The subsequent displacement of iodine with potassium cyanide led to (*S*)-**19** or (*R*)-**20**, and the final hydrolysis gave Boc-β^3^-homoAla-OH (*S*)-**21** or (*R*)-**22** (Scheme 2). The risk of racemization was excluded by HPLC on a chiral column and polarimetry, as reported [43].

**Scheme 3:**
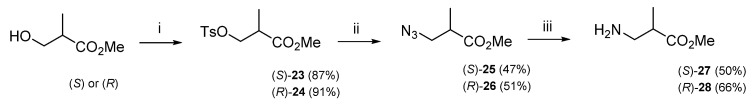
Synthetic procedure for (*S*)- or (*R*)-β^2^-homoAla-OMe. Reagent and conditions: (i) TsCl, TEA, DCM, 0 °C, rt, overnight; (ii) NaN_3_, DMF, 50 °C, 16 h; (iii) H_2_, Pd/C, EtOH, rt, 12 h.

The enantiopure model residue (*S*)- or (*R*)-β^2^-Ala was synthesized as methyl ester by a modified version of a multi-step procedure previously reported by Lee et al [44]. Tosylation of (*S*)- or (*R*)-β-hydroxy ester provided (*S*)-**23** or (*R*)-**24** in good yield, 87% and 91%, respectively (Scheme 3). The tosylate was treated with NaN_3_ in N,N-dimethylformamide (DMF) to afford the corresponding azide (*S*)-**25** or (*R*)-**26**. Finally, the catalytic reduction of the crude azide with H_2_ and the catalytic amount of Pd/C gave the primary amine (*S*)-**27** or (*R*)-**28**.

**Scheme 4:**
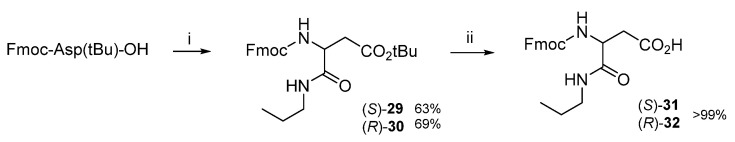
Synthetic procedure for (*S*)- or (*R*)-Fmoc-β^3^-isoAsp(NHPr)-OH. Reagents and conditions: (i) n-propylamine, EDC.HCl/HOBt/TEA, DCM/DMF, rt, 3 h; (ii) TFA/DCM (1:1), rt, 1 h.

As anticipated (Figure 2), to introduce a central β^3^-homo residue possessing an extra anchoring point, we selected (*S*)- or (*R*)-isoaspartate (isoAsp). The carboxylic acid of isoAsp can be utilized to connected useful linkers by straightforward reactions (Figure 2). To mimic such linkers, the α carboxylic group of (*S*)- or (*R*)-Fmoc-Asp(OtBu)-OH was reacted with a simple *n*-propylamine using 1-ethyl-3-(3-dimethylaminopropyl)carbodiimide**^.^**HCl (EDC)/hydroxybenzotriazole (HOBt)/triethylamine (TEA) as activating agents, giving the fully protected **29** or **30**. Subsequent *t*Bu-deprotection with trifluoroacetic acid (TFA) afforded Fmoc-isoAsp(NHPr)-OH (*S*)-**31** or (*R*)-**32** (Scheme 4 and Supplementary Materials).

Finally, we selected a β^2^-amino acid core carrying an amino group side chain, i.e., (*S*)- or (*R*)-2,3-diaminopropionic acid (Dap) (Figure 2). For the preparation of this residue, we opted for the Hofmann rearrangement of *N*-protected (*S*)- or (*R*)-Asn in solid phase, giving the corresponding Dap peptidyl resin (Scheme 8). In this case, the amide side chain of Asn was intended as a convenient protected precursor of the β-amino group of Dap, orthogonal to Fmoc.

#### 3.1.2. Synthesis of Hybrid Peptides

**Scheme 5:**
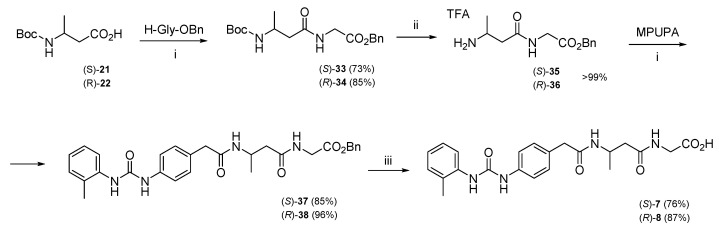
Synthetic procedure for MPUPA-β^3^-homoAla-Gly-OH, (*S*)-**7**, (*R*)-**8**. Reagent and conditions: (i) EDC/HOBt, TEA, DCM/DMF, rt, 12 h; (ii) 25% TFA/DCM, rt, 1 h; (iii) H_2_, Pd/C, EtOH, rt, 12 h.

The hybrid sequences **7** and **8** were prepared in solution. Boc-β^3^-Ala (*S*)-**21** or (*R*)-**22** was coupled with H-Gly-OBn.TFA in the presence of the coupling agents EDC/HOBt/TEA. The resulting Boc-dipeptide **33** or **34** was treated with TFA and the deprotected dipeptide **35** or **36** was N-capped with MPUPA using the same activating agents. Catalytic hydrogenation of the benzyl ester **37** or **38** afforded (*S*)-**7** or (*R*)-**8** in good yield after purification by semipreparative reversed phase (RP)-HPLC (**7**, 76%; **8**, 87%; Scheme 5).

For the preparation of (*S*)-**9** or (*R*)-**10**, β^2^-homoAla-OMe (*S*)-**27** or (*R*)-**28** was coupled in solution with MPUPA in the presence of 2-(1H-benzotriazole-1-yl)-1,1,3,3-tetramethylaminium tetrafluoroborate (TBTU)/HOBt/N,N-diisopropyl ethylamine (DIPEA). Hydrolysis of the resulting ester **40** or **41** with LiOH gave the carboxylic acid (*S*)-**42** or (*R*)-**43** that was coupled with H-Gly-OtBu**^.^**HCl salt, under the same coupling conditions previously described. The crude mixture was purified by RP HPLC (general methods) giving (*S*)-**44** or (*R*)-**45** in acceptable yields (**4**, 50%; **5**, 55%). 

**Scheme 6:**
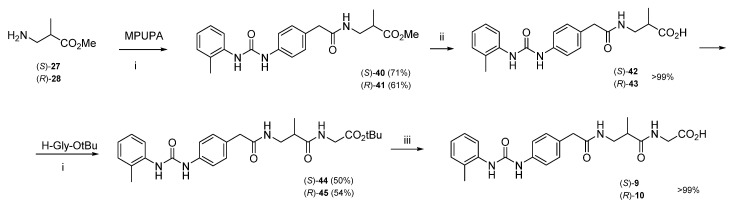
Synthetic procedure for MPUPA-β^2^-homoAla-Gly-OH, (*S*)-**9**, (*R*)-**10**. Reagent and conditions: (i) TBTU, HOBt, DIPEA, DCM/DMF, rt, 12 h; (ii) LiOH, THF/H_2_O, 0 °C, rt, 2 h, then 0.1 N HCl; (iii) TFA/DCM, rt, 1 h.

Finally, deprotection of the *t*Bu group with TFA gave the final peptidomimetic (*S*)-**9** or (*R*)-**10** in almost quantitative yield (Scheme 6) and excellent purity (95 and 94%) after precipitation from ether.

**Scheme 7:**
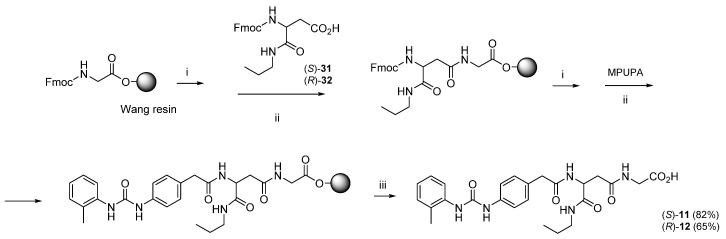
Synthetic procedure for MPUPA-isoAsp(NHPr)-Gly-OH, (*S*)-**11**, (*R*)-**12**. Reagent and conditions: (i) 20% Pip/DMF, rt, 10 min (×2); (ii) DCC, HOBt, DCM/DMF, rt, 3 h; (iii) TFA/H_2_O/TIPS/PhOH (78:10:10:2 *v*/*v*/*v*/*v*), rt, 2.5 h.

Peptide (*S*)-**11** or (*R*)-**12** was prepared by SPPS on a Wang resin preloaded with Fmoc-Gly (Scheme 7). The Fmoc group was removed with 20% piperidine (Pip) in DMF. Coupling with Fmoc-isoAsp(NHPr)-OH (*S*)-**31** or (*R*)-**32** was carried out with dicyclohexylcarbodiimide/(DCC/HOBt) in DCM/DMF. After Fmoc deprotection with Pip/DMF, and subsequent coupling with MPUPA, under the same conditions described above, the cleavage of the peptide was accomplished by treatment of the peptidyl resin with TFA in the presence of scavengers. The crude product (*S*)-**11** or (*R*)-**12** was precipitated from ice-cold Et_2_O and collected in acceptable yields by centrifuge (Scheme 7), and finally purified by semipreparative RP-HPLC.

**Scheme 8:**
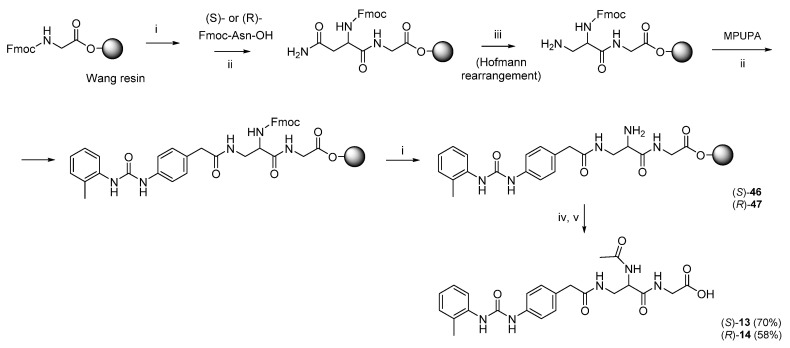
Synthetic procedure for MPUPA-Dap(Ac)-Gly-OH, (*S*)-**13**, (*R*)-**14**. Reagents and conditions: (i) 20% Pip/DMF, rt, 10 min (×2); (ii) DCC/HOBt, DCM/DMF, rt, 3 h; (iii) PIFA, pyridine, THF/DMF/H_2_O (2:2:1), rt, 3 h; (iv) Ac_2_O, pyridine, DCM, rt, 1 h; (v) TFA/H_2_O/TIPS/PhOH (78:10:10.2 *v*/*v*/*v*/*v*), rt, 2.5 h.

Finally, (*S*)-**13** or (*R*)-**14** were assembled by SPPS on a Wang resin preloaded with Fmoc-Gly (Scheme 8). After removal of Fmoc group with 20% Pip/DMF, Fmoc protected (*S*)- or (*R*)-Asn-OH was activated for coupling with (*S*)- or (*R*)-Fmoc-Asn-OH in the presence of DCC/HOBt. The Hofmann rearrangement of the primary amide of Asn into the corresponding amine was performed on resin using PhI(OCOCF_3_)_2_ (PIFA) and pyridine in DMF/H_2_O [45]. The resulting Nα-Fmoc-Dap residue was coupled at the β-amino group with MPUPA under the same conditions described above. 

Then, Fmoc deprotection of Dap gave the peptidyl resin (*S*)-**46** or (*R*)-**47**. The subsequent acetylation with Ac_2_O and pyridine completed the linear sequence. Peptide cleavage was accomplished by treatment of the resin with TFA and scavengers (Scheme 8). The crude product **13** or **14** was precipitated from ice-cold Et_2_O, collected by centrifuge, and purified by semipreparative RP-HPLC (general methods).

In general, after synthesis the crude peptides of all sequences were purified by semipreparative RP HPLC over a C18 column (general methods), using H_2_O/acetonitrile mixtures containing 0.1% trifluoroacetic acid; purity was assessed to be >95% by RP HPLC; chemical identity was confirmed by ESI-MS, ^1^H, ^13^C and 2D gCosy NMR spectroscopy.

### 3.2. Biochemical Cheracterization

#### 3.2.1. Potency and Selectivity of Hybrid Peptides for Human α4β1 Integrin as Measured by Inhibition of Integrin-Mediated Cell Adhesion to Endogenous Ligands

The ability of the reference compounds BIO1211 and **6**, and of the new hybrid peptides **7**–**14**, to modulate integrin-mediated cell adhesion to the α4β1 endogenous ligand FN, was measured by cell adhesion assays. Integrin ligands able to inhibit cell adhesion promoted by the endogenous ligand were referred to as antagonist. Jurkat cells, an immortalized cell line of human T lymphocytes, are often utilized as prototypic α4β1 integrin-expressing cells [22,46]. The reference compound BIO1211 and the hybrid peptide **6** prevented cell adhesion with nanomolar IC_50_ values (Table 1), as previously described [22,32].

Concerning the hybrid peptidomimetics, the IC_50_ values strongly varied depending on the type of β-residue, on (*S*)- or (*R*)-configuration, as well as on the nature of the central β-residue side chain. Compound **7**, containing (*S*)-β^3^-Ala, showed a very modest micromolar IC_50_ (4.06 µM), and the enantiomer **8** was defined as not active (i.e., IC_50_ > 5 µM). As for the peptides containing β^2^-Ala, both enantiomers (*S*)-**9** and (*R*)-**10** were not able to modulate α4β1 integrin-mediated cell adhesion to a significant extent. This result was somewhat unexpected, since the parent peptide DS-70 (**6**), containing (*S*)-β^2^-Pro, had a noteworthy nanomolar, IC_50_ (4.3 ± 1.7 nM). Plausibly, the rigid structure of the pyrrolidine scaffold conferred to the entire peptide a constrained, more appropriate bioactive conformation [47]. 

The experiments performed on the remaining α/β hybrid peptides **11**–**14** gave highly contrasting results as compared to the model peptides **7**–**10** containing β^3^- or β^2^-Ala (Table 1). Peptide **12**, containing the (*R*)-isoAsp(NHPr), showed much higher potency with respect to the (*S*) enantiomer **11**, IC_50_ = 9.8 nM vs. IC_50_ > 5000 nM, respectively. Topologically, the (*R*)-configured β^3^-amino acid isoAsp is consistent with (*S*)-β^3^-Ala, since the side chains of both residues, when observed in the fully extended conformation, remain below the molecular plane (Figure 2). On the other hand, peptide **13** containing the β^2^-residue (*S*)-Dap showed a meaningful IC_50_ in the submicromolar range, while the enantiomer **14** was inactive. Apparently, the amide groups at the side chains of **12** and **13** strongly improved the compound’s ability to modulate cell adhesion mediated by α4β1 integrin, since the topologically corresponding model peptides carrying a simple methyl side chain were practically inactive (Table 1).

These experiments pointed at **12** and **13** as effective antagonist ligands of α4β1 integrin, potentially useful for therapeutic applications in inflammatory and allergic pathologies. These opportunities will be fully developed in due course. In addition, for the presence of functionalized side chains, **12** and **13** can be conveniently exploited in the design and preparation of biomaterials capable of reacting to α4β1 integrin cells, provided that these compounds display sufficient stability in physiological conditions. 

#### 3.2.2. In Vitro Enzymatic Stability of **12**, **13**

The stability of the hybrid peptides MPUPA-(*R*)-isoAsp(NHPr)-Gly-OH (**12**) and MPUPA-(*S*)-Dap(Ac)-Gly-OH (**13**) was estimated in vitro by incubation in mouse serum at 37 °C, in comparison with BIO1211. The incubation mixture was sampled during 180 min and the quantity of intact peptides was determined by HPLC analysis. As shown in the Supplementary Materials, BIO1211 was rapidly hydrolyzed, since only about 10% of the initial amount was still present after 1 h. On the other hand, the peptides **12** and **13** appeared to be much more stable under the same conditions, and after 3 h the amount of the two peptides was around 80% and 91%, respectively. These data provide evidence that the presence of the β-amino acid markedly increased the enzymatic stability.

The comparatively higher stability of **13** vs. **12** was not completely unexpected. Indeed, the (*S*)-β^2^-amino acid Dap is topologically consistent with an (*R*)-α-amino acid (Figure 2). Hence, the higher stability of **13** could correlate with the presence of the β-residue as well as with the unnatural display of its side chain. 

### 3.3. Design of Linkable α4β1 Integrin Ligands

**Scheme 9:**
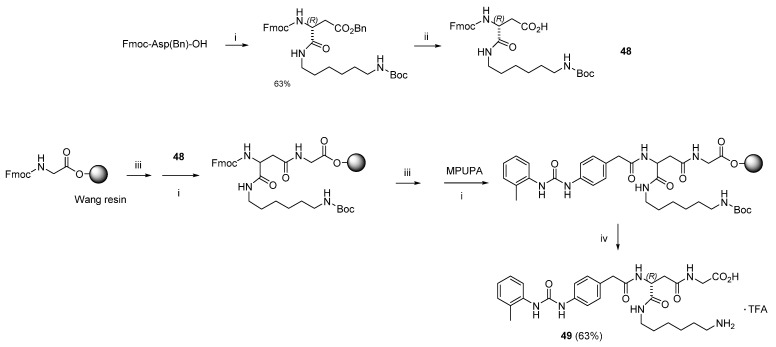
Synthetic procedure for MPUPA-(*R*)-isoAsp(Hda)-Gly-OH (**49**). Reagents and conditions: (i) DCC/HOBt, DCM/DMF, rt, 3 h; (ii) H_2_, Pd/C, EtOH, rt, 12 h; (iii) 20% Pip/DMF, rt, 10 min (×2); (iv) TFA/H_2_O/TIPS/PhOH (78:10:10:2 *v*/*v*/*v*/*v*), rt, 2.5 h.

**Scheme 10:**
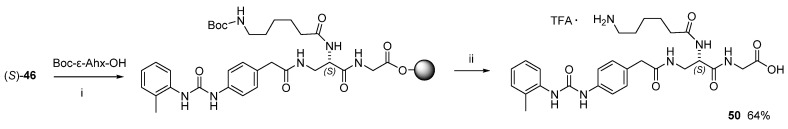
Synthetic procedure for MPUPA-(*S*)-Dap(Ahx)-Gly-OH (**48**). Reagents and conditions: (i) DCC/HOBt, DCM/DMF, rt, 3 h; (ii) TFA/H_2_O/TIPS/PhOH (78:10:10:2 *v*/*v*/*v*/*v*), rt, 2.5 h.

The α4β1 integrin ligands MPUPA-(*R*)-isoAsp(NHPr)-Gly-OH (**12**) and MPUPA-Dap(Ac)-Gly-OH (**13**) were selected for the biofunctionalization of microstructured monolayers to provide surfaces potentially capable of recognizing and binding α4β1 integrin-expressing cells. Although the hybrid peptide **12** was by far the most potent integrin ligand (Table 1), we utilized **13** also, since the latter appeared to be slightly more stable, as estimated by incubating the peptides in mouse serum (see above). Hence, we modified the structures of **12** and **13** (Scheme 9 and Scheme 10) by introduction of effective linkers for anchoring onto the surfaces via urea linkages [17]).

For the modification of peptide **12**, (*R*)-Fmoc-Asp(OBn)-OH was reacted with Boc mono-protected 1,6-hexanediamine (Boc-Hda, Supplementary Materials) using EDC/HOBt/TEA as activating agents. Hydrogenolysis gave Fmoc-isoAsp(Boc-Hda)-OH **48** (Scheme 9). Subsequently, **48** was utilized for the SPPS of the peptide MPUPA-(*R*)-isoAsp(Hda)-Gly-OH.TFA (**49**), under the same conditions described for the preparation of **12**. The treatment with TFA in the presence of scavengers allowed at the same time peptide cleavage and the removal of the Boc group at Hda (Scheme 9).

Peptide **13** was modified by substituting the acetyl group with 6-aminohexanoic acid (Ahx), giving the peptide MPUPA-Dap(Ahx)-Gly-OH.TFA (**50**). In detail, the MPUPA-(*S*)-Dap-Gly peptidyl resin **46**, prepared as described in Scheme 8, was functionalized at the free α-amino group with Boc-Ahx-OH, activated with DCC/HOBt. Eventually, the treatment of the Boc-protected peptidyl resin with TFA/scavengers allowed to cleave the peptide and to deprotect the amino group of Ahx (Scheme 10).

### 3.4. Preparation of Microstructured Surfaces and Functionalization with Peptides **49**, **50**

As prototypic microstructured substrates to be functionalized by the peptides, we opted for monolayers of flat zeolite L crystals, positioned over round (ø 12 mm) silica plates [17,48]. These crystals can be conveniently employed for the construction of cell growth surfaces, being characterized by a large surface area and expedient superficial functionalization [49]. The plates were treated with aminopropyltriethoxysilane (APTES), giving aminopropyl (AP)-plates (Supplementary Materials). Flat zeolite L crystals, between 600 and 1000 nm wide and about 250 nm in height, prepared as reported in the literature (Supplementary Materials) [48], were loaded for visualization with the dye N,N^0^-bis(2,6-dimethylphenyl) perylene-3,4,9,10-tetracarboxylic acid diimide (DXP) [50]. Subsequently, the crystals were treated with 3-(isocyano)-propyltriethoxysilane (ICPTES, Supplementary Materials).

**Scheme 11:**
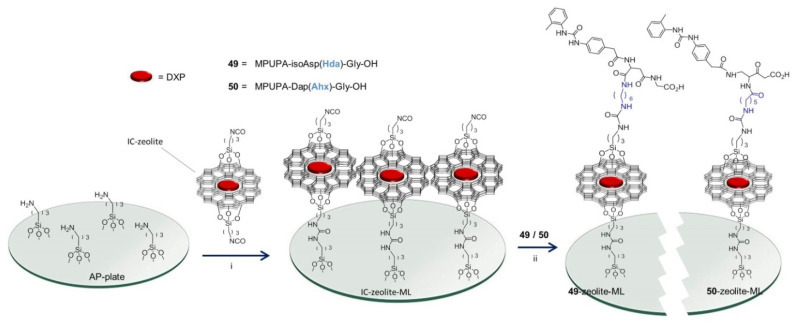
Synthetic procedure for peptidyl–zeolite MLs. Reagents and conditions: (i) toluene, sonication, rt, 30 min; (ii) TEA, DMF, 40 °C, 3 h. Hda, 1,6-hexanediamine; Ahx, 6-aminohexanoic acid; AP, aminopropyl; IC, isocyano; ML, monolayer.

**Figure 3:**
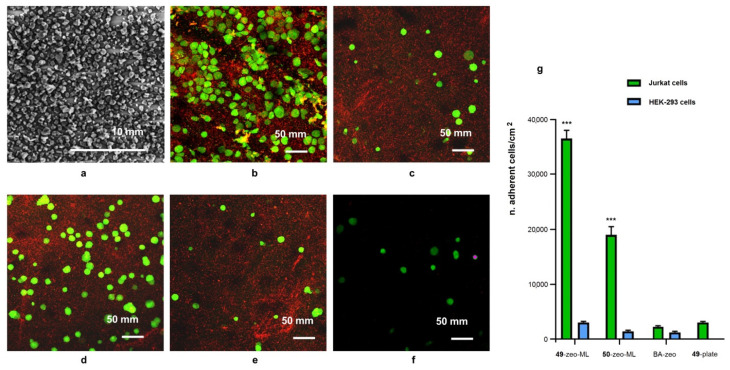
(**a**) SEM image of IC–zeolite ML. Cell adhesion to peptide monolayers: (**b**–**f**) confocal microscopy images of monolayers after 15 min incubation at 37 °C with 5 × 10^5^ cells; (**b**) Jurkat cells adhered to **49**–zeolite ML; (**c**) HEK-293 cells adhered to **49**–zeolite ML; (**d**) Jurkat cells on **50**–zeolite ML; (**e**) Jurkat cells on BA–zeolite ML; (**f**) Jurkat cells on **49**-plate; (**g**) Number of adhered cells/cm^2^ (analyzed area: 0.0025 cm^2^); data are shown as mean ± SD of three independent experiments. *** *p* < 0.001 vs. HEK-293 seeded onto peptide-zolite ML. Cells are visualized in green, while zeolites are visualized in red.

To immobilize the resulting isocyano (IC)–zeolites, the AP-plates were immersed in a suspension of IC–zeolites, giving IC–zeolite monolayers (IC–zeolite MLs, Figure 3a), connected by highly stable urea linkages (Scheme 11). 

The efficacy of each subsequent functionalization of the native zeolites with organic molecules was assessed by the increase in the percentage values of C(1s) and N(1s) as determined by X-ray photoelectron spectrometry (XPS) analysis (Table 2 and Supplementary Materials). In particular, the IC–zeolite MLs showed C(1s) and N(1s) values (%) similar to the values obtained for the unbound zeolites. Thermogravimetric analyses (TGA) verified a higher weight loss for the functionalized zeolites as compared to the native zeolites (Supplementary Materials).

Eventually, the IC–zeolite MLs were immersed in a solution of peptide **49** or **50** in DMF in the presence of TEA to give the monolayers **49**–zeolite MLs or **50**–zeolite MLs (Scheme 11). XPS analysis confirmed the large increase of C(1s)/N(1s) percentages.

To challenge the stability under physiological conditions prior to performing the cell adhesion experiments, peptide–zeolite MLs were immersed at rt for 24 h in mouse serum diluted in sterile milli-Q water. Then, the monolayers were washed with distilled water. Subsequent XPS analysis showed the same atomic percent values, confirming the stability of the peptidic functionalization (not shown).

Alternatively, IC–zeolite MLs were functionalized with n-butylamine (BA) to afford BA–zeolite MLs, designed as peptide-negative references. XPS analysis showed a inferior increase of C(1s)/N(1s) for BA–zeolite MLs as compared to peptide–zeolite MLs (Table 2 and Supplementary Materials).

As a further control, silica plates were directly functionalized with peptide **49** without interposing the crystals (see Discussion). To the purpose, bare silica plates were reacted with ICPTES, and the IC-plates were treated with peptide **49**, under the same conditions utilized for the preparation of **49**–zeolite MLs. XPS analysis of the resulting **49**-plates was suggestive of a moderate functionalization (Table 2 and Supplementary Materials).

### 3.5. Cell Adhesion Experiments

To measure cell adhesion to peptidyl–zeolite MLs, experiments were performed by seeding either Jurkat cells, mainly expressing α4β1 integrin [19,31], or HEK-293 control cells not expressing α4 integrin, which are not expected to adhere upon rapid incubation [51]. Cells were stained with a green fluorescent dye (CMFDA) and seeded on the peptide monolayers. After a very brief incubation of 15 min, the non-adherent cells were washed away, while the attached cells were fixed with paraformaldehyde.

Consistent with the nM IC_50_ of the parent peptide **12**, confocal microscopy analysis (Figure 3b) revealed that **49**–zeolite MLs had the highest number of adherent Jurkat cells (3.6 ± 0.16 × 10^4^ cells/cm, analyzed area: 0.0025 cm^2^; Figure 3g), while the number of adherent integrin-negative control HEK-293 cells was far lower (2.8 ± 0.2 × 10^3^ cells/cm) (Figure 3c,g). The monolayers **50**–zeolite MLs (Figure 3d) showed a reduced but still remarkable number of adherent Jurkat cells (1.9 ± 0.15 × 10^4^ cells/cm; Figure 3g).

The peptide-negative reference BA–zeolite ML (Figure 3e) showed almost negligible adhesion of Jurkat cells (2.6 ± 0.2 × 10^3^ cells/cm, Figure 3g). Longer incubation times led to a moderate but scarcely reproducible increase in the number of adherent cells. Finally, confocal microscopy showed few adherent Jurkat cells (3.0 ± 0.2 × 10^3^ cells/cm) after seeding onto **49**-plates deprived of zeolite crystals (Figure 3f).

## 4. Discussion

Most inflammatory diseases, including allergies and asthma, are characterized by α4β1 integrin-mediated extravasation and exaggerated accumulation of leukocytes [52]. Indeed, the activation of lymphocytes promotes both integrin clustering and their switch to a higher affinity conformation, and these events rapidly strengthen cell interaction with the endogenous ligands overexpressed on the vascular surface of activated endothelial tissue [19]. Leukocyte migration is also typical of autoimmune diseases characterized by chronic inflammation, e.g., rheumatoid arthritis, autoimmune encephalomyelitis, and multiple sclerosis [53].

In this scenario, the development of materials capable of interacting specifically with lymphocytes by specific recognition of α4β1 integrins could find practical applications in diagnostics (see Introduction). As a proof of concept, herein we describe the preparation of monolayers of zeolites coated with novel ligands inspired from the potent α4β1 integrin antagonist BIO1211 [32], stably bonded to the crystals via urea linkages. Generally, the practicability of peptide–material conjugates is questioned mainly because the peptides grafted onto biomaterial surfaces might be rapidly hydrolyzed by serum proteins in vivo, thus nullifying the functionalization [9,10,54,55]. In point of fact, BIO1211 was found to have poor stability under physiological conditions [33,34]. As a consequence, we took into consideration the use of more stable peptidomimetic ligands. Of notice, there are at present no stable ligands available to specifically bind to α4β1 integrin while simultaneously carrying suitable linkers for bioconjugation. This is in net contrast to the class of RGD-binding integrins, for which a variety of suitable ligands has been proposed (Figure 1), including the well-known cyclopeptide c(RGDfK) (**1**) [15], conveniently equipped with a flexible aminoalkyl side chain.

Moving from the hybrid α/β peptide DS70 (**6**, Figure 1) [22], we designed a mini-library of very simple mimetics of BIO1211 composed of α- and β-residues, i.e., the peptides **7**–**14**, upon substitution of the β-Pro scaffold with the model residues β^2^- or β^3^-Ala, or by their respective functionalized analogues Dap and isoAsp, in (*S*)- or (*R*)-configuration (Figure 2). 

The ability of these compounds to modulate α4β1 integrin-mediated cell adhesion was assayed in vitro in comparison with the reference compound BIO1211. The best results were observed for MPUPA-(*R*)-isoAsp(NHPr)-Gly-OH (**12**) and MPUPA-Dap(Ac)-Gly-OH (**13**) (Table 1). The comparison between these peptides and the model peptides containing β-Ala suggested that the presence of amide groups at the side chains of **12** and **13** may significantly contribute to a compound’s ability to inhibit α4β1-mediated cell adhesion. The detailed study of the effects exerted by the different substituents on ligand–receptor interactions is beyond the scope of this work. Further studies by molecular modeling and molecular docking are currently in progress in our group. 

For the presence of functionalized side chains, which can be equipped with taggable or linkable side chains, **12** and **13** were selected for the preparation of biomaterials. To the purpose, the peptides were modified by introducing suitable linkers, giving MPUPA-(*S*)-isoAsp(Hda)-Gly-OH (**49**) and MPUPA-(*S*)-Dap(Ahx)-Gly-OH (**50**), respectively (Scheme 9 and Scheme 10). Thereafter, the monolayers **49**–zeolite ML and **50**–zeolite MLs were expediently prepared by stably anchoring the peptides onto MLs of zeolite L crystals mounted over silica plates (Scheme 10).

α4β1 Expressing Jurkat cells or integrin-negative HEK-293 cells were seeded onto the peptide MLs. Consistent with the high affinity of the parent **12** for α4β1 integrin, **49**–zeolite MLs showed the highest number of adherent Jurkat cells, as revealed by confocal microscopy analysis. Both peptide MLs showed an excellent discrimination of Jurkat cells with respect to reference HEK-293 cells (Figure 3g). The latter are known to express significant levels of β1 integrin but very low levels of α4 integrin [31,51]. As expected, Jurkat cells showed negligible adhesion onto MLs deprived of the peptide (BA–zeolite ML).

Interestingly enough, Jurkat cells showed little adhesion to monolayers of peptides directly anchored to the silica plates (**49**-plates). Plausibly, the nanostructured surface of zeolite L crystals significantly fosters cell adhesion. Due to their flat shape and peculiar microstructure, during the formation of the monolayers the nanocrystals tend to adopt a clear orientation, with the cavities perpendicular to the support surface (Scheme 11) [56]. As a consequence, the paved surface appears interspersed with a regular alternation of solid parts and cavities, so that the bonded ligands maintain a regular spacing from each other, forming a well-organized biofunctionalized surface. Integrin-mediated adhesion is known to be regulated by multiple features of the adhesive surface, including topography and spacing between the ligands [57]. Plausibly, the resulting nanostructured surface might enhance cell recognition due to the regular positioning of the biomolecules, correlated in turn to the regular distance between adjacent channels in the nanocrystals.

## 5. Conclusions

On the basis of the results above discussed, zeolite MLs, functionalized with hybrid α/β-peptidomimetic ligands of α4β1 integrin, may be exploited for preparing functionalized biomaterials for a wide range of medical applications, from therapy to diagnosis, including the possibility of developing diagnostic devices capable of detecting desired cells in biological fluids obtainable from patients in a noninvasive way. In perspective, more functional assays will be performed to better investigate the effect of the hybrid ligands on transmigration, chemotaxis, and intracellular signaling in inflammatory cells, including neutrophils and monocytes. Moreover, the peptide-coated materials might be utilized as model systems to study in detail the mechanisms of integrin-mediated adhesion and signaling using different types of inflammatory cells.

## Figures and Tables

**Table 1:** Inhibition of α4β1 integrin-mediated Jurkat cell adhesion ^a^ to plates coated with the endogenous ligand FN (10 μg/mL) by the reference compounds BIO1211 and DS-70 (**6**), and by the new hybrid peptides **7**–**14**.

Compd β-Residue Type	Structure	IC_50_ (nM) ^b^
BIO1211-	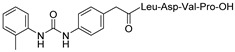	5.5 ± 4.0 ^c^
DS70 **6** β^2^	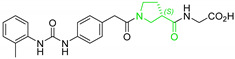	4.3 ± 1.7 ^c^
MA97 **7** β^3^	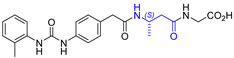	4060 ± 780
MA99 **8** β^3^	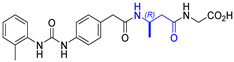	>5000
MA192 **9** β^2^	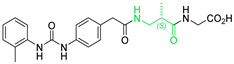	>5000
MA199 **10** β^2^	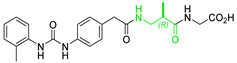	>5000
MA62 **11** β^3^	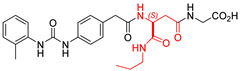	>5000
MA158 **12** β^3^	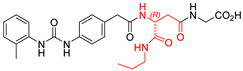	9.8 ± 2.1
MA28 **13** β^2^	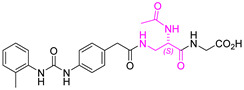	236 ± 47
MA29 **14** β^2^	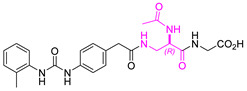	>5000

^a^ In a cell-based assay, the adhesion of Jurkat cells to an immobilized endogenous ligand (FN, 10 μg/mL) was measured. ^b^ Three independent experiments were run in quadruplicate. Data are expressed as means ± SD. ^c^ [22].

**Table 2:** XPS analysis results (atomic %) for zeolite L materials and the peptide monolayers; mean ± SE of three to four analyses. Bare zeolites showed very low C(1s) and N(1s) signals; these percentage values increased significantly and consistently to the subsequent functionalization steps.

Material	O(1s)	N(1s)	C(1s)	K(2p)	Si(2p)	Al(2p)	O(1s)
Zeolite L	58.0 ± 1.1	0.8 ± 0.2	5.8 ± 0.9	6.9 ± 1.2	21.5 ± 0.2	7.0 ± 0.7	58.0 ± 1.1
DXP–zeolite	44.9 ± 3.8	2.1 ± 0.3	21.1 ± 4.0	5.5 ± 0.8	18.5 ± 2.0	7.9 ± 3.0	44.9 ± 3.8
IC–zeolite	29.5 ± 4.0	5.0 ± 1.0	32.0 ± 6.0	4.0 ± 1.0	17.9 ± 3.0	11.6 ± 2.0	29.5 ± 4.0
IC–zeolite ML	35.9 ± 1.2	5.3 ± 2.0	31.0 ± 3.2	1.9 ± 1.0	17.9 ± 3.1	8.0 ± 1.5	35.9 ± 1.2
**49**–zeolite ML	31.2 ± 2.0	8.3 ± 1.6	44.4 ± 4.5	0.8 ± 0.4	13.5 ± 2.0	1.8 ± 0.5	31.2 ± 2.0
**50**–zeolite ML	32.2 ± 2.3	8.8 ± 2.1	43.0 ± 2.0	0.5 ± 0.2	13.9 ± 1.9	1.6 ± 0.3	32.2 ± 2.3
BA–zeolite ML	37.8 ± 3.0	4.9 ± 1.1	32.2 ± 3.8	1.2 ± 0.3	17.9 ± 2.5	6.0 ± 0.4	37.8 ± 3.0
IC-plate ^1^	57.4 ± 3.0	0.9 ± 0.4	5.0 ± 1.9	4.9 ± 1.6	19.9 ± 2.0	1.9 ± 0.9	57.4 ± 3.0
**49**-plate ^1^	53.1 ± 4.0	2.0 ± 0.6	10.1 ± 3.0	4.2 ± 1.0	18.1 ± 2.8	1.6 ± 0.6	53.1 ± 4.0

^1^ The rest being B, Na, Zn, and Ti.

## Data Availability

Not applicable.

## Supplementary Materials

The following are available online at https://www.mdpi.com/article/10.3390/biomedicines9111737/s1: Experimental procedures; Figure S1: Stability of BIO1211, **12**, and **13**, in mouse serum; Figure S2: SEM image of zeolite L crystals, TGA analyses, XPS analyses.
