# Supplementary material for: Design of α/β-Hybrid Peptide Ligands of α4β1 Integrin Equipped with a Linkable Side Chain for Chemoselective Biofunctionalization of Microstructured Materials
Source: Biomedicines. 2021 Nov 21;9(11):1737. doi: 10.3390/biomedicines9111737 (PMC8615975; doi:10.3390/biomedicines9111737)
Supplement: Supplementary file 1 [file biomedicines-09-01737-s001.zip › biomedicines-1440834-supplementary.pdf]

Supporting Information

Design of α/β-hybrid peptide ligands of $\alpha4\beta1$ integrin equipped with a linkable side chain for chemoselective biofunctionalization of microstructured materials

Michele Anselmi[1,§], Monica Baiula[2,§], Federica Santino[1], Jumwei Zhao[1], Santi Spampinato[2], Natalia Calonghi[2], and Luca Gentilucci[1,*]

[1] *Department of Chemistry "G. Ciamician", University of Bologna, via Selmi 2, 40126-Bologna, Italy;*

[2] *Department of Pharmacy and Biotechnology, University of Bologna, via Irnerio 48, 40126-Bologna, Italy*

[§] *Equally contributed to the work.*

** Correspondence: luca.gentilucci@unibo.it; Tel.: +39 0512099570*

Table of Contents

Experimental procedures 2

Figure S1: Stability of BIO1211, **12**, and **13**, in mouse serum 4

Figure S2: SEM image of zeolite L crystals, TGA analyses, XPS analyses 5

MPUPA. o-Tolyl isocyanate (6.6 mmol) was added dropwise to a suspension of 4-aminophenylacetic acid (6.6 mmol) in DMF (6 mL). The reaction mixture was stirred at rt for 3 h under inert atmosphere. Then, the mixture was diluted with EtOAc (40 mL) and the precipitate (77%) was collected as a whitish solid by filtration. ESI-MS *m/z* calcd. for $[C_{16}H_{17}N_2O_3]^+$: 285.1, found: 285.2 $[M+H]^+$.

tBu-(1-Hydroxypropan-2-yl)carbamate (*S*)-**15**, (*R*)-**16**. To a stirred solution of N-methylmorpholine (NMM, 1.1 mmol) and Boc-(*S*)- or (*R*)-Ala-OH (1.0 mmol) in dry THF (5 mL), ethylchloroformate (1.1 mmol) was added dropwise at 0 °C. After 15 min, the solution was filtered and NMM.HCl salt was washed with THF (3 × 5 mL). The filtrates were collected and a suspension of $NaBH_4$ (1.3 mmol) in H_2O (4 mL) was then added dropwise at 0°C while stirring. The reaction proceeded at rt, and after 20 min the solvent was evaporated at reduced pressure. The residue was re-dissolved in EtOAc (50 mL) and the mixture was washed with H_2O (5 mL) and brine (5 mL), then the organic layer was dried over Na_2SO_4. Evaporation of the solvent at reduced pressure afforded the product (*S*)-**15** (98%) or (*R*)-**16** (89%), as a yellow oil, which was used without further purifications. ^{1}H NMR (400 MHz, $CDCl_3$) δ 4.67 (br s, 1H, NH), 3.78-3.60 (m, 1H, Hα), 3.50 (dd, *J* = 13.8, 7.0 Hz, 1H, Hβ), 3.26 (dd, *J* = 12.4, 7.0 Hz, 1H, Hβ), 1.43 (s, 9H, tBu), 1.27 (d, *J* = 6.8 Hz, 3H, CH_3). ESI-MS *m/z* calcd. for $[C_8H_{18}NO_3]^+$: 176.1, found: 176.2 $[M+H]^+$.

tBu-(1-Cyanopropan-2-yl)carbamate (*S*)-**19**, (*R*)-**20**. To a stirred solution of triphenylphosphine (1.3 mmol) in dry dichloromethane (DCM, 5 mL), I_2 (1.3 mmol) was added at rt under inert atmosphere. After 15 min, imidazole (2.5 mmol) was also added and the mixture was stirred for additional 15 min. Then, a solution of the Boc-protected amino alcohol (*S*)-**15** or (*R*)-1**6** (1.0 mmol) in dry DCM (5 mL) was added and the mixture was heated at reflux until consumption of the starting material for about 3 h; reaction progress was monitored by thin layer chromatography (TLC). Then the mixture was cooled, diluted with DCM (40 mL), and washed with 10% $Na_2S_2O_5$ (20 mL) and brine (2 × 10 mL). The organic layer was dried over Na_2SO_4 and the solvent was evaporated at reduce pressure to give (*S*)-**17** or (*R*)-**18**.

The crude iodide (*S*)-**17** or (*R*)-**18** was then dissolved in dry DMSO (30 ml) and solid KCN (2.0 mmol) was added in one portion. The mixture was stirred under N_2 at 60°C for about 4 h (TLC monitoring). The mixture was then poured into water, and extracted with EtOAc (40 mL), and the organic layer was finally washed with brine (2 × 20 mL). The organic layer was dried over Na_2SO_4, then the solvent was evaporated at reduced pressure. The crude residue was purified by flash chromatography over silica gel (eluent: cyclohexane/EtOAc 80:20) to give (*S*)-**19** (40 % over two steps) or (*R*)-**20** (52 % over two steps), as a white solid. ^{1}H NMR (400 MHz, $CDCl_3$) δ 4.71 (br s, 1H, NH), 4.00 – 3.88 (m, 1H, Hα), 2.72 (dd, *J* = 17.6, 5.2 Hz, 1H, Hβ), 2.52 (dd, *J* = 16.8, 4.0 Hz, 1H, Hβ), 1.43 (s, 9H, tBu 1.31 (d, *J* = 6.8 Hz, 3H, CH_3). ESI-MS *m/z* calcd. for $[C_9H_{17}N_2O_2]^+$: 185.1, found: 185.2 $[M+H]^+$.

Boc-β^3-HomoAla-OH (*S*)-**21**, (*R*)-**22**. The amino nitrile (*S*)-**19** or (*R*)-**20** (1.0 mmol), was dissolved in 1M KOH/EtOH/ (1:1 v/v, 5 mL). The mixture was heated at 90°C while stirring for 3 h. The mixture was cooled to rt and EtOH was distilled at reduced pressure. The residue was cooled to 0°C and 1M $KHSO_4$ was added dropwise until pH was 2-3. Then, the product was extracted with EtOAc (3 × 20 mL). The combined organic layers were washed with H_2O (2 × 10 mL) and brine (10 mL). The organic layer was dried over Na_2SO_4, filtered, and concentrated at reduced pressure to give (*S*)-**21** (60%) or (*R*)-**22** (50%) respectively. Racemization was excluded by chiral HPLC analysis performed on an HP1100 instrument with UV–VIS

detector, using a Chiralpak IC column (25 × 0.46 cm, mobile phase: 1:1 n-hexane/2-propanol, flow rate 0.8 mL/min). (*S*)-**21** $[\alpha]_D^{20}$ = -17° (c = 0.1, $CHCl_3$); (*R*)-**22** $[\alpha]_D^{20}$ = +18° (c = 0.08, $CHCl_3$). ^{1}H-NMR (400 MHz, DMSO-d_6) δ 7.60 (br d, 1H, NH), 4.00-3.88 (m, 1H, Hα), 2.42 (dd, *J* = 17.4, 5.6 Hz, 1H, Hβ), 2.32 (dd, *J* = 16.6, 4.8 Hz, 1H, Hβ), 1.43 (s, 9H, tBu), 1.31 (d, *J* = 6.8 Hz, 3H, CH_3). ESI-MS *m/z* calcd. for $[C_9H_{18}NO_4]^+$: 204.1, found: 204.2 $[M+H]^+$, 226.1 $[M+Na]^+$.

Methyl 2-methyl-3-(tosyloxy)propanoate (*S*)-**23**, (*R*)-**24**. To a stirred solution of (*S*)- or (*R*)-methyl 3-hydroxy-2 methylpropanoate (1.0 mmol) in dry DCM, trimethylamine (TEA, 1.2 mmol) and a solution of tosyl chloride (1.2 mmol) in dry DCM (5 mL) were sequentially added dropwise at 0 °C. The mixture was stirred overnight at rt, then it was diluted with DCM (20 mL) and washed with H_2O (3 × 5 mL). The organic layer was dried over Na_2SO_4, filtered and concentrated at reduced pressure. The crude material was purified by flash chromatography over silica gel (eluent: cyclohexane/EtOAc 80:10; *Rf* = 0.18 in cyclohexane/EtOAc 60:10) to give the respective (*S*)-**23** (87%) or (*R*)-**24** (91%), as a colourless oil.

Methyl 3-amino-2-methylpropanoate (*S*)-**27**, (*R*)-**28**. A mixture of (*S*)-**23** or (*R*)-**24** (1.8 mmol) and NaN_3 (2.0 mmol) in dry N,N-dimethylformamide (DMF, 5 mL) was stirred at 50°C for 16 h. After cooling, the mixture was diluted with EtOAc (20 mL), and the organic layer was washed with H_2O (3 × 5 mL). The collected organic layer was dried over Na_2SO_4 and evaporated at reduced pressure to afford the crude (*S*)-**25** (47%) or (*R*)-**26** (51%), which was used for the next step without further purification.

The crude azide **25** or **26** was hydrogenated in the presence of a catalytic amount of Pd/C (10% w/w) overnight at rt. The catalyst was removed by filtration through Celite®, and the filtrates were evaporated to afford the respective crude (*S*)-**27** (50%) or (*R*)-**28** (66%) which was triturated in Et_2O and collected by filtration. ESI-MS *m/z* calcd. for $[C_5H_{12}NO_2]^+$: 118.1, found: 118.3 $[M+H]^+$.

*Fmoc-isoAsp(propylamine)-OH (S)-**31** or (R)-**32***. In line with the general coupling procedure, Fmoc-(*L*)-Asp(OtBu)-OH or Fmoc-(*D*)-Asp(OtBu)-OH (1.22 mmol) was activated with EDC/HOBt (1.22 mmol each) for 10 min, then *n*-propylamine (1.22 mmol) was added, and the mixture was stirred under inert atmosphere at rt for 3 h. After the workup, the residues were purified by flash chromatography over silica gel (gradient eluent Cy/EtOAc 80:20 to 70:30) to afford (*S*)-**29** (350 mg, 63%) and (*R*)-**30** (380 mg, 69%), respectively. *Rf* = 0.36 (Cy/EtOAc 60:40). ^{1}H-NMR (400 MHz, $CDCl_3$) δ 7.78 (d, *J* = 7.5 Hz, 2H, ArH), 7.60 (d, *J* = 7.5 Hz, 2H, ArH), 7.42 (t, *J* = 7.5 Hz, 2H, ArH), 7.33 (t, *J* = 7.4 Hz, 2H, ArH), 6.47 (br.s, 1H, AspNH), 5.97 (d, *J* = 8.0 Hz, 1H, propylNH), 4.45 (d, *J* = 6.8 Hz, 2H, $FmocCH_2$), 4.23 (dd, *J* = 7.2, 6.8 Hz, 1H, AspHα), 3.22 (q, *J* = 5.9 Hz, 2H, $propylCH_2$), 2.93 (dd, *J* = 17.0, 3.8 Hz, 1H, AspHβ), 2.60 (dd, *J* = 17.2, 6.8 Hz, 1H, AspHβ), 1.57-1.48 (m, 2H, $propylCH_2$), 1.46 (s, 9H, *t*Bu), 0.91 (t, *J* = 7.4 Hz, 3H, $propylCH_3$). ESI-MS *m/z* calcd. for $[C_{26}H_{33}N_2O_5]^+$: 453.2, found: 453.0 $[M+H]^+$.

Removal of *t*Bu-protecting group from **29** or **30** was performed as described in the general procedure (B). Pure intermediates (*S*)-**31** and (*R*)-**32** were obtained in quantitative yield and directly used in the next step without further purifications. ESI-MS *m/z* calcd. for $[C_{22}H_{25}N_2O_5]^+$: 397.2, found: 397.0 $[M+H]^+$.

*Fmoc-(R)-isoAsp(Boc-Hda)-OH **48**.* (*R*)-Fmoc-Asp(OBn)-OH (0.8 mmol) was reacted with tert-butyl (6-aminohexyl)carbamate (Boc-Hda, 0.9 mmol) using the activating agents EDC/HOBt/TEA (0.4/0.4/0.8 mmol), under the same conditions described above. After the usual work up, the resulting crude Fmoc-isoAsp(Boc-

Hda)-OBn (0.7 mmol, 87%) was utilized for the next step without purification. ESI-MS *m/z* calcd. for $[C_{37}H_{46}N_3O_7]^+$: 644.3, found: 644.4 $[M+H]^+$. Catalytic hydrogenation of Fmoc-isoAsp(Boc-Hda)-OBn (0.7 mmol) over 10% Pd/C (w/w) as described above gave the Fmoc-protected isoaspartate **48** in almost quantitative yield (95%). ESI-MS *m/z* calcd. for $[C_{30}H_{40}N_3O_7]^+$: 554.3, found: 554.4 $[M+H]^+$.

Boc 1,6-hexanediamine (Boc-Hda). A 0.5 M solution of Boc_2O (1 mmol) in chloroform was added during 2h to a 0.25 M solution of 1,6-hexanediamine (5 mmol) in chloroform at 0°C. The mixture was stirred for 8h at rt and filtered. The filtrate was concentrated at reduced pressure, the residue was diluted with EtOAc (30 mL), and the organic layer was washed with brine (2 x 5 mL). The organic layer was dried over Na_2SO_4, and the solvent was removed at reduced pressure giving Boc-Had (80%), utilized without further purification. ESI-MS *m/z* calcd. for $[C_{11}H_{25}N_2O_2]^+$: 217.2, found: 217.3 $[M+H]^+$.

IC-zeolites. The Zeolites (300 mg) and N,N^0-bis(2,6-dimethylphenyl) perylene -3,4,9,10-tetracarboxylic acid diimide (DXP, 6.2 mg) were kept overnight under high vacuum. Then, the solid mixture was heated at 300°C in a sealed flask into a rotary furnace. The flask was broken and the powder was washed with butan-1-ol and centrifuged (30 min, 40 krcf), until the supernatant ceased UV emission. The DXP-loaded zeolites (50 mg) were suspended in a mixture of toluene (5 mL) and TEA (0.2 mL), and the precipitate was dissolved by sonication. Then, ICPTES (0.8 mL) was added, and the mixture was sonicated for 3 h. The suspension was cooled to rt and centrifuged for 20 min (40 krcf). The precipitate was dispersed three times in toluene by sonication, and collected by centrifugation.

AP-plates. Glass plates were placed into a 100 mL flask, and cleaned by treatment with 3:1 H_2SO_4/H_2O_2 (10 mL) at 100°C for 1 h. The plates were rinsed with bidistilled water (3 × 30 mL) and ethanol (3 × 20 mL), and finally dried by blowing dry N_2. The plates were aligned onto a Teflon support, and the rack was immersed into a mixture of APTES (0.8 mL) and TEA (0.2 mL) in toluene (30 mL). The system was heated overnight at 115°C. Subsequently, the plates were washed with toluene (5 mL) and EtOH (5 mL), and dried under N_2 flow.

IC-zeolite-MLs. The AP-plates were immersed into a suspension of IC-zeolites in toluene (1 mg/mL, 2 mL), and sonicated for 30 min, and then the plates were washed with toluene (3 x 5 mL) and air dried.

Figure S1

Stability of BIO1211, **12**, and **13**, in mouse serum. Samples were removed from the incubation solution at 0, 0.15, 0.5, 1.0, 2.0, 3.0 h and peptide stability was determined using an RP-HPLC ESI-MS analysis (described in the General Methods). Values are presented as mean ± SD (n = 4).

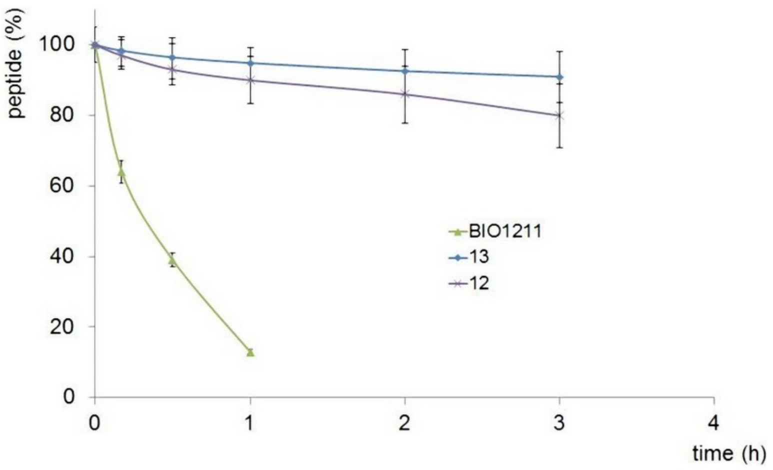

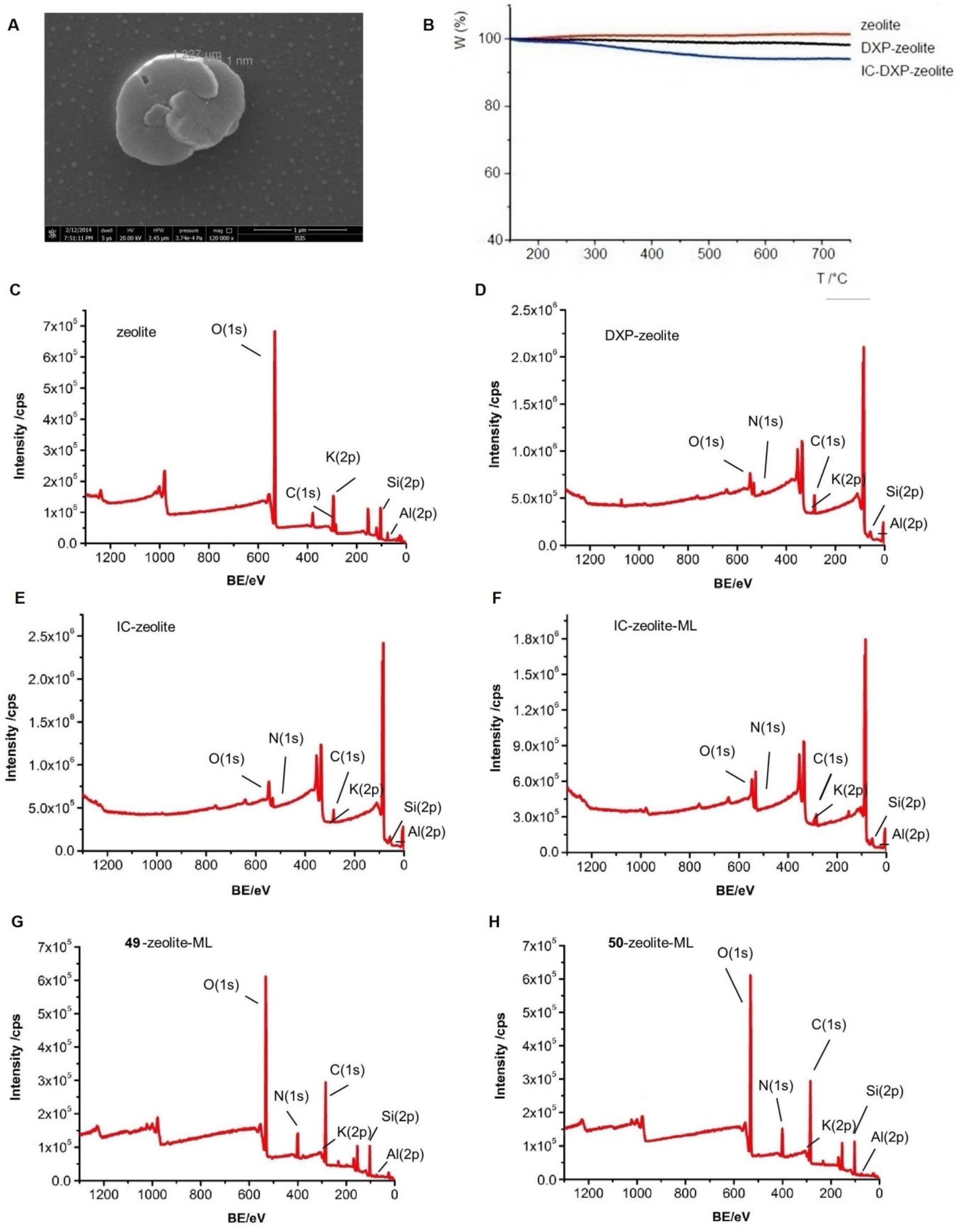

Figure S2. (**A**) SEM image of disk-shaped zeolite L crystals; scale bar = 1000 nm. (**B**) TGA analysis of the pristine and the derivatized zeolite L materials, normalized after removing the contribution of the water present in the pristine zeolites. XPS analyses for (**C**) the pristine, (**D**) the DXP-loaded, (**E**) the IC-derivatized zeolite L crystals. XPS analyses for monolayers of zeolite L crystals functionalized with (**F**) ICPTES (IC), (**G**) peptide **49**, (**H**) peptide **50**.
